# Effectiveness of progesterone-primed ovarian stimulation in assisted reproductive technology: a systematic review and meta-analysis

**DOI:** 10.1007/s00404-020-05939-y

**Published:** 2021-01-12

**Authors:** Ling Cui, Yonghong Lin, Fang Wang, Chen Chen

**Affiliations:** 1 Department of Reproduction and Infertility, Chengdu Women’s and Children’s Central Hospital, School of Medicine, University of Electronic Science and Technology of China, Chengdu, 611731 China; 2 School of Biomedical Science, University of Queensland, St Lucia, Brisbane, QLD Australia

**Keywords:** Ovarian stimulation, Assisted reproductive technology, Controlled ovarian stimulation, Clinical pregnancy rate, Live birth rate

## Abstract

**Purpose:**

Progestin-primed ovarian stimulation (PPOS) is a new ovarian stimulation protocol that has been used over the last decade to enhance reproductive function. The purpose of this study is to evaluate whether PPOS is as effective as conventional protocols (without GnRHa downregulation).

**Method:**

Search terms included “medroxyprogesterone”, “dydrogesterone”, “progestin-primed ovarian stimulation”, “PPOS”, “oocyte retrieval”, “in vitro fertilization”, “IVF”, “ICSI”, “ART”, and “reproductive”. The selection criteria were nonrandomized studies and randomized controlled studies. For data collection and analysis, the Review Manager software, Newcastle–Ottowa Quality Assessment Scale and GRADE approach were used.

**Results:**

The clinical pregnancy rates were not significantly different in either RCTs or NRCTs [RR 0.96, 95% CI (0.69–1.33), *I*^2^ = 71%, *P* = 0.81]; [RR 0.99, 95% CI (0.83–1.17), *I*^2^ = 38%, *P* = 0.88]. The live birth rates of RCTs and NRCTs did not differ [RCT: RR 1.08, 95% CI (0.74, 1.57), *I*^2^ = 66%, *P* = 0.69; NRCT: OR 1.03 95% CI 0.84–1.26), *I*^2^ = 50%, *P* = 0.79]. The PPOS protocol had a lower rate of OHSS [RR 0.52, 95% CI (0.36–0.75), *I*^2^ = 0%, *P* = 0.0006]. The secondary results showed that compared to the control protocol, the endometrium was thicker [95% CI (0.00–0.78), *I*^2^ = 0%, *P* = 0.05], the number of obtained embryos was higher [95% CI (0.04–0.65), *I*^2^ = 17%, *P* = 0.03] and more hMG was needed [in NRCT: 95% CI (307.44, 572.73), *I*^2^ = 0%, *P* < 0.00001] with the PPOS protocol.

**Conclusion:**

The PPOS protocol produces more obtained embryos and a thicker endometrium than the control protocol, with a lower rate of OHSS and an equal live birth rate. The PPOS protocol could be a safe option as a personalized protocol for infertile patients.

**Trial registration:**

Registration at PROSPERO: CRD42020176577.

## Introduction

Progestin-primed ovarian stimulation (PPOS) was proposed by the Yanping Kuang M.D. group in 2015 [1]. Oral administration of exogenous progesterone (P), such as medroxyprogesterone acetate (MPA) and dydrogesterone (DYG) [2–5], beginning in the early follicular phase is used with gonadotropin during controlled ovarian stimulation (COS) [defined by The International Committee for Monitoring Assisted Reproductive Technology (ICMART) and the World Health Organization (WHO)] [6] in IVF/ICSI treatments. PPOS can effectively prevent the activation and transmission phases of oestradiol (E2)-induced LH surges and thus serves as an alternative to conventional treatment with GnRH analogs. Prior studies have shown that the PPOS protocol with medroxyprogesterone acetate (MPA) produces competent oocytes/embryos and achieves comparable pregnancy outcomes to those of GnRH antagonist protocols [3, 4, 7–11], as well as short-term protocols [12, 13] and mild stimulation protocols [5] (see Table 1). Coupled with the application of frozen-thawed embryo transfer (FET) and the dual trigger of GnRH agonist with low-dose hCG, the PPOS protocol also allows for nearly complete avoidance of OHSS occurrence [14, 15], since all the embryo transfers after PPOS are frozen. There are many clinical studies on PPOS protocol use in infertile women, including women who have normal ovarian function, PCOS [4, 15], poor ovarian response [7, 9], who are of advanced maternal age [5], having endometriosis [11] and donated oocytes [10]. The reported findings are variable; some studies have shown better live birth outcomes, while others showed no difference. The crucial clinical aspects of IVF protocols are efficacy and safety. Some studies have shown that the PPOS protocol may be cost-effective compared with the GnRH antagonist in planned freeze-only cycles, such as in preimplantation genetic testing or fertility preservation [11, 16]. These results are very consistent with our clinical observations, but we still need more solid evidence.

**Table 1:** Characteristics of included studies

Author (year)	Country	Type of study	Participants	IVF protocol	Intervention
Eftekhar et al. (2019) [4]	Iran	RCT^1^	With PCOS aged between 18 and 40 years (*n* = 60/each)	PPOS^2^ vs GnRH^3^ antagonist protocol	PPOS: rFSH^4^(Cinnal-f Cinnagen, Iran)for injection, DYG^5^for oral progesterone Antagonist protocol: cetrotide (Merck-Serono Germany) for injection
Chen et al. (2019) [7]	China	RCT	Participants with poor responders mean age was 35 (*n* = 170/each)	PPOS vs GnRH antagonist protocol	PPOS: hMG^6^for injection, MPA^7^ for oral progesterone Antagonist protocol: cetrotide + hMG
Wen et al. (2018) [12]	China	RCT	PPOS (MPA 10 mg) and short-term protocol with maximum age was 35 years (*n* = 31/each)	PPOS vs short-term protocol	PPOS: hMG (Lizhu Pharmaceutical Trading Co., Zhuhai, China) for injection, medroxyprogesterone acetate (MPA, Xianju Pharma, Zhejiang, China) for oral progesterone Short-term protocol: triptorelin (Huilin, Germany) + hMG
Begueria et al. (2019) [8]	Spain	RCT	Women between 18 and 35 years (*n* = 91/each)	PPOS vs GnRH antagonist protocol	PPOS: rFSH (Gonal-F Merck, Madrid, Spain) for injection, MPA (Progevera Pfizer, Spain) for oral progesterone Antagonist protocol: ganirelix (Orgalutran, Merck Sharp and Dohme Limited, UK)
Wang et al. (2016) [15]	China	RCT	Patients with PCOS. age 18–39 years (*n* = 60/each)	PPOS vs short-term protocol	PPOS: hMG (Anhui Fengyuan Pharma ceutical Co, China) for injection, MPA (Beijing Zhong Xin Pharmaceutical, China) for oral progesterone Short-term protocol: hMG for injection
Iwami et al. (2018) [3]	Japan	Prospective nonrandomized controlled study	Age younger than 41 years 125 in study group and 126 in control group	PPOS vs GnRH antagonist protocol	PPOS: hMG (Teizo, ASKA Pharmaceutical Co., Ltd., Tokyo, Japan) for injection, DYG (Duphaston, Abbott Healthcare, Tokyo, Japan) for oral progesterone Antagonist protocol: ganirelix (Ganirelix MSD, Tokyo,Japan) or Cetrotide (EMD-Serono, Tokyo, Japan)
Wang et al. (2018) [13]	China	Retrospective cohort study	1107 cycles from the PPOS protocol and 969 cycles from the GnRH-a short protocol	PPOS vs short-term protocol	PPOS: hMG (Anhui Fengyuan Pharmaceutical Co., Ltd., Hefei, China) for injection, utrogestan (Laboratories Besins International, Paris, France) for oral progesterone Short-term protocol: cetrorelix (Decapeptyl, Ferring Pharmaceuticals, Germany)
Huang et al. (2019) [9]	China	Retrospective cohort study	Poor ovarian responders 63 cycles from the PPOS protocol and 123 cycles from the GnRH-a short protocol	PPOS vs GnRH antagonist protocol	PPOS: hMG (Anhui Fengyuan Pharmaceutical Co, China) for injection, MPA (Beijing Zhong Xin Pharmaceutical, China) Antagonist protocol: cetrorelix (Decapeptyl, Ferring Pharmaceuticals, Germany)
Peng et al. (2019) [5]	China	retrospective cohort study	Women with ages ≥ 40 years. 122 cycles mild stimulation group and PPOS group (47 cycles)	PPOS vs mild stimulation protocol	PPOS: hMG (Lizhu Pharmaceutical Trading Co., Zhuhai, China) for injection, DYG (Duphaston; Abbott Biologicals B.V., Netherlands) for oral progesterone The mild stimulation protocol: CC^8^(Codal Synto Ltd., Cyprus) + hMG
Yildiz et al. (2019) [10]	Turkey	Retrospective cohort study	103 donors, mean age 25 years 49 PPOS group 54 control group	PPOS vs GnRH antagonist protocol	PPOS: rFSH (Gonal F, MerckvSerono, Switzerland)) for injection, MPA (Tarlusal, Deva) for oral progesterone Antagonist protocol: (Cetrotide, Merck Serono)
Mathieu d’Argent et al. (2020) [11]	France	Retrospective cohort study	Age < 40 years, with endometriosis (n = 54/each)	PPOS vs GnRH antagonist protocol	PPOS: rFSH for injection, desogestrel for oral progesterone Antagonist protocol: (Ganirelix, orgalutran MSD France)

^1^RCT: randomized controlled trial

^2^PPOS: progestin-primed ovarian stimulation

^3^GnRH: gonadotropin-releasing hormone

^4^rFSH: recombinant human follicle stimulating hormone

^5^DYG: dydrogesterone

^6^hMG: human menopausal gonadotropin

^7^MPA: medroxyprogesterone acetate

^8^CC: clomiphene citrate

It is questionable whether PPOS has the same effect and is safer than conventional IVF protocols. The purpose of this systematic review was to investigate whether PPOS for the treatment of infertile patients achieved pregnancy outcomes that were the same as or better than those of conventional protocols (any COS protocol without gonadotrophin-releasing hormone agonist (GnRHa) downregulation). This work will hopefully provide statistical evidence for clinicians on PPOS use in the treatment of infertility.

## Methods

### Criteria for considering studies for this review

We performed a pairwise meta-analysis.

### Types of studies

We included intervention studies in the form of randomized controlled trials and nonrandomized controlled trials that compared progestin-primed ovarian stimulation to other protocols.

### Types of participants

Participants suffering from infertility.

### Types of interventions

One of the interventions for IVF was PPOS, and the control interventions included the GnRH agonist protocol, as well as the short-term protocol and mild stimulation protocol (details of protocols are shown in Table 1).

### Types of outcome measures

Primary outcomes:

1. Clinical pregnancy rate [6]
2. Live birth rate [6]
3. Incidence of OHSS [6]

Secondary outcomes:

1. Duration of stimulation
2. Dose of gonadotrophin for injection
3. Progestin values on trigger day (ng/ml)
4. Number of retrieved oocytes
5. Number of MII oocytes
6. Number of obtained embryos
7. Total cycle cancelation
8. Endometrial thickness

## Data collection and analysis

### Selection of studies

The titles and abstracts of articles were screened by two independent researchers (LC, FW) to be included or excluded. Any disagreement between the two as to which studies to include was resolved by discussion. A third author (YHL) would evaluate records when there was any unsolvable disagreement.

### Data collection process

Data were extracted by one reviewer (LC), and checked by a second (FW). For each included study, the information collected included study design, methods, setting and time period, information about the participants (eligibility criteria), and drop-outs; interventions and outcomes, including clinical pregnancy rate, live birth rate, incidence of OHSS, duration of stimulation, dose of gonadotrophin for injection, progestin values on trigger day (ng/ml), number of retrieved oocytes, number of MII oocytes (mature oocytes), number of obtained embryos, total cycle cancelation, and endometrial thickness.

### Search methods for identification of studies

This study was based on the PRISMA guidelines for systemic review and meta-analysis [17]. The electronic databases used were MEDLINE, EMBASE, and the Cochrane Library from 2010 to 13th March 2020 without limitation of region, language, or publication type. Specific strategies for electronic search at the database used a combination of (MeSH): ((((((medroxyprogesterone) or Dydrogesterone)) or progestin-primed ovarian stimulation) or PPOS)) and ((((oocyte retrieval rate) or IVF) or ICSI) or ART). The following keywords “medroxyprogesterone”, “dydrogesterone”, “progestin-primed ovarian stimulation”, “PPOS”, “oocyte retrieval”, “IVF”, “ICSI”, “ART”, and “reproductive” were used in the search. Intervention studies including prospective controlled study, retrospective cohort study, nonrandomized studies with comparison groups (NRCTs), and randomized controlled trial were included. The inventions of the control group included short-term protocol, GnRH antagonist protocol, and mild stimulation protocols (any cos protocol without GnRHa downregulation). The strategies for electronic search at the database used a combination of (MeSH) ((((((medroxyprogesterone) or Dydrogesterone)) or progestin-primed ovarian stimulation) or PPOS)) and ((((oocyte retrieval rate) or IVF) or ICSI) or ART).

We excluded the following studies: (1) self-controlled study; (2) books, conferences, review articles, editorial, notes, thesis, case series, letters, posters, and case reports; (3) unreliable extracted data, overlapped datasets, and paragraphs of only abstract available.

## Assessment of risk of bias in individual studies

### Quality of studies

The Cochrane collaboration tools were used to assess the risk of bias in randomized controlled trials [18]. The Cochrane Collaboration risk of bias tool includes random sequence generation (selection bias), allocation sequence concealment (selection bias), blinding of participants and personnel performance bias (performance bias), blinding of outcome assessment (detection bias), incomplete outcome data (attrition bias), selective reporting (reporting bias), and other bias. The reviewers rated the quality of the included studies as low risk, unclear risk or high risk.

Newcastle–Ottawa Scale (NOS) was used to assess the quality of nonrandomized controlled studies in meta-analyses [19]. The NOS is useful, reliable, complementary tools for appraising methodological quality of medical education research [20]. The NOS contains eight items. The items are categorized into three dimensions including selection, comparability, and outcomes of studies. The NOS ranges from zero to nine stars as follows: selection of the study group (up to 4 stars/points), comparability of cohorts (up to 2 stars/points), and ascertainment of outcome (up to 3 stars/points). High-quality studies achieve more than seven stars, medium-quality studies between four and six stars, and poor-quality studies less than four stars.

### Data synthesis

All data were entered into the analysis system (Review Manager, version 5.2). We used the risk ratio (RR) and 95% confidence intervals (CIs) for variables with dichotomous data for RCTs and odds ratios (ORs) for nonrandomized studies. For these variables, the weighted summary RR was calculated using the Mantel–Haenszel method. For continuous data, the mean difference (MD) was calculated and corrected according to the sample bias.

We constructed ‘Summary of findings’ tables using GRADE-pro [21]. We summarized and graded the certainty of the evidence for critical outcomes (clinical pregnancy rate, live birth rate, OHSS, duration of stimulation, dose of gonadotrophin for injection, number of retrieved oocytes, number of obtained embryos, and endometrial thickness).

### Subgroup analysis and investigation of heterogeneity

Higgins *I*^2^ values [22] were used to assess statistical heterogeneity between studies and values of *I*^2^ ≤ 25% which were indicative of low heterogeneity.

We used a fixed-effect model in the analysis, as our results were all homogeneous according to the chi-squared test and *I*^2^ ≤ 50%. The random-effect model was used in the analysis, our results were all homogeneous according to the chi-squared test, and 50% ≤ *I*^2^ ≤ 70% was taken to indicate substantial statistical heterogeneity. If the chi-squared test result and *I*^2^ were ≥ 70%, where the heterogeneity was too large and not suitable for combined analysis, we performed a subgroup analysis. The effectiveness of HMG versus recombinant FSH in women undergoing ovarian stimulation for IVF/ICSI demonstrated a significant difference in the live birth rate [23, 24]. We performed subgroup analysis for clinical pregnancy rate (primary outcome), live birth rate (primary outcome), and dose of sex hormones for injection (secondary outcome) considering the different types of sex hormones for injection (rFSH or hMG) according to clinical experience.

### Sensitivity analysis

For outcomes such as the number of MII oocytes, we examined the sensitivity versus risk of bias (by excluding one study [12] with unclear risks of bias from the analysis of selection bias, performance bias, detection bias, attrition bias, selective reporting, and reporting bias). We also assessed the outcome of gonadotrophin subgroup (hMG) sensitivity to risk of bias (by excluding one study [12] with unclear risks of bias from the analysis of selection bias, performance bias, detection bias, attrition bias, selective reporting, and reporting bias and one study [15] with a large difference in the mean ± SD (2072.5 ± 467.86 vs. 1501.25 ± 68.18).

## Results

### Results of the search

**Fig. 1:**
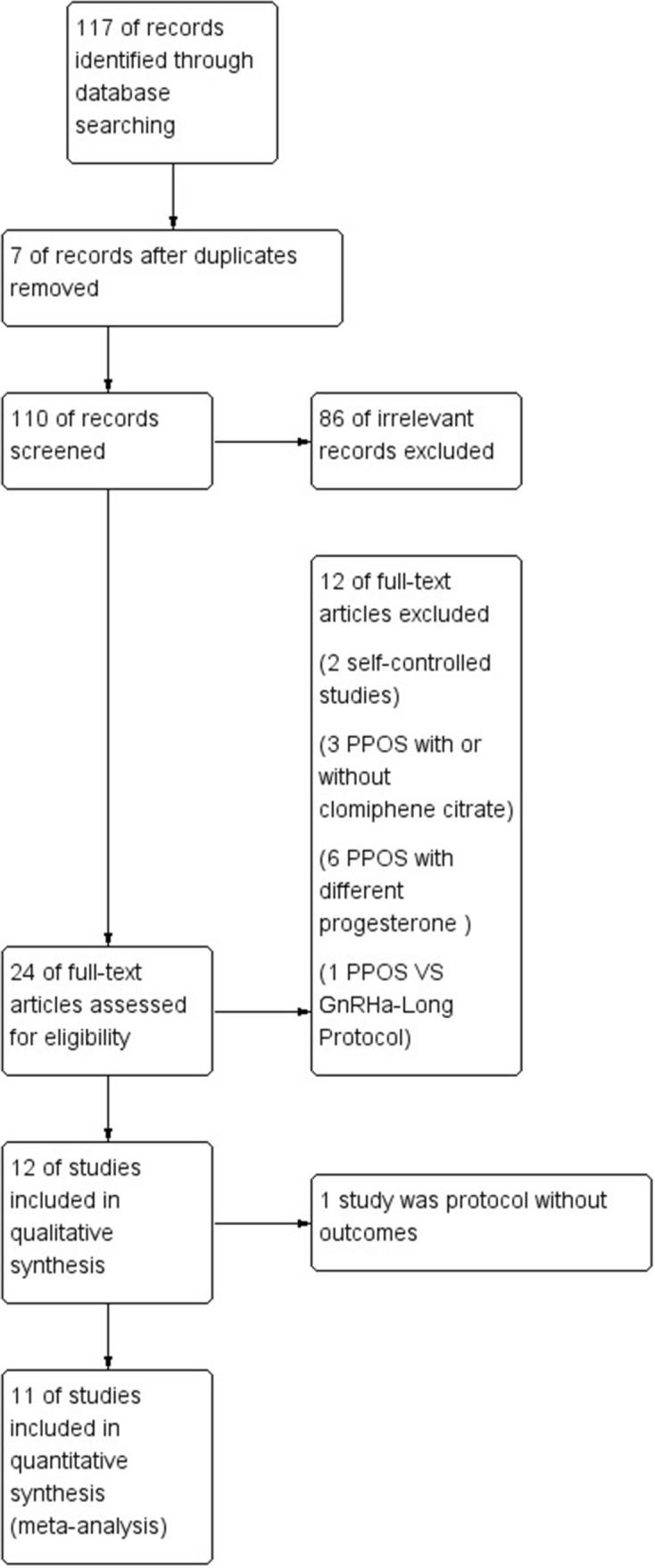
PRISMA flow diagram of study selection for the systematic review and meta-analysis

We identified a total of 117 records from the electronic database searches. Deduplication and removal of all irrelevant records were performed. After the titles and abstracts were screened, 86 irrelevant records were excluded. Of the remaining 24 studies, we excluded 13 records. Details of the selection process for studies are summarized in the PRISMA flow diagram (Fig. 1). There were five RCTs, one nonrandomized study and five retrospective cohort studies (Table 1).

### Description of populations and interventions

Table 1 provides brief details of populations and interventions. Two RCTs [4, 15] included PCOS participants, and the studies by Chen et al. [7] and Huang et al. [9] included participants with poor responders. Wen et al. [12] and Begueria et al. [8] included participants with a maximum age of 35 years. Iwami et al. [3] and Mathieu d’Argent et al. [11] included participants with maximum ages of 41 and 40 years. Peng et al. [5] included participants aged ≥ 40 years. Yildiz et al. [10] included participants with donor oocytes.

### Quality of studies

**Fig. 2:**
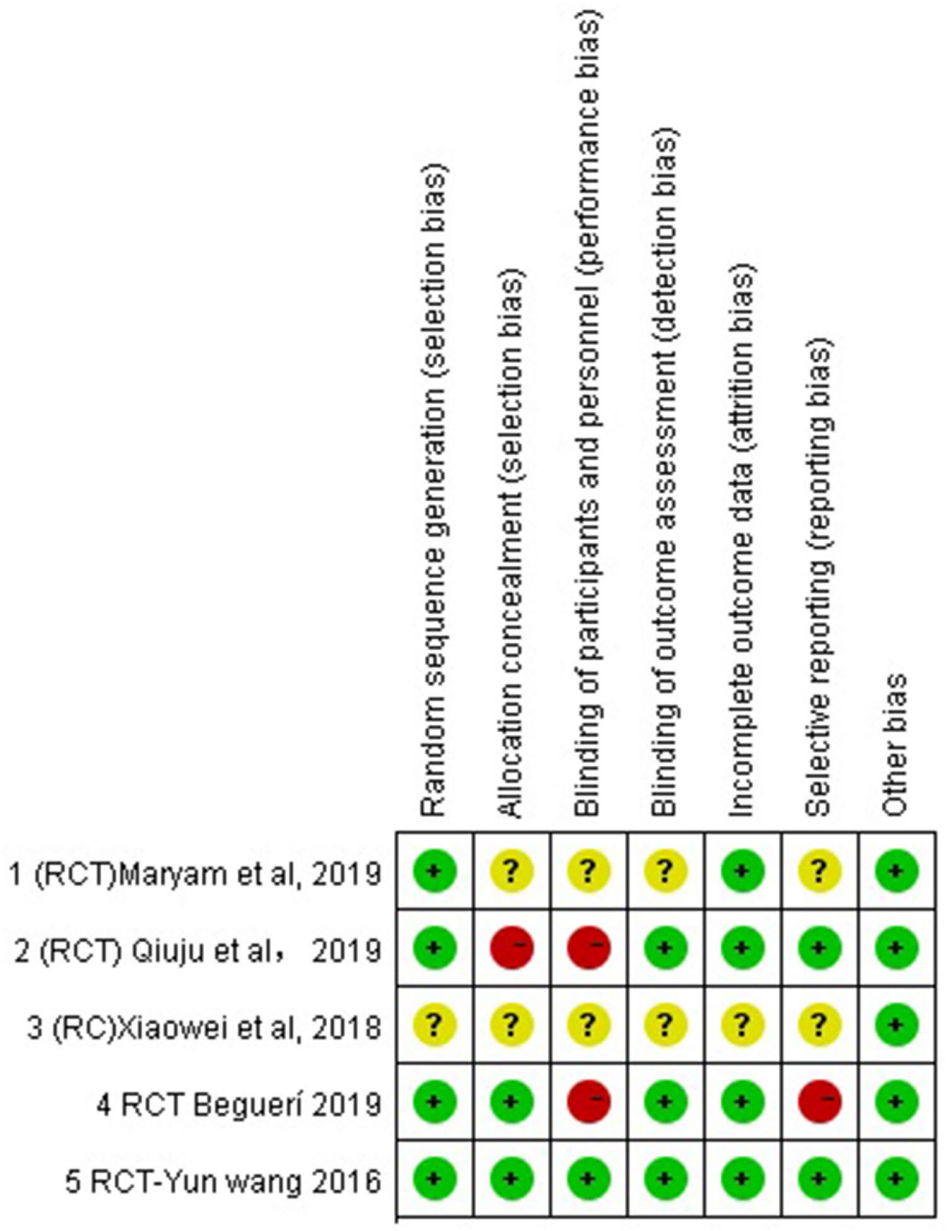
Risk of bias assessment for the randomized studies

The quality of the studies included varied widely. Randomized control trials (RCTs) were assessed for their methodological quality using the Cochrane Risk of Bias Tool. The full details of the risk of bias assessment for the randomized studies are given below (Fig. 2). Three of five RCTs had four or five out of seven domains with a low risk of bias, but one study [12] had six unclear risks of bias. Three of six nonrandomized studies achieved seven stars and were judged as high quality. The other three achieved four to six stars and were judged to be of medium quality. Full details of the Newcastle–Ottawa Scale (NOS) scores for the nonrandomized studies are provided in Table 2.

**Table 2:** Newcastle–Ottawa risk of bias for included NRCTs

Author (year)	Selection of study groups score	Comparability of groups score	Outcome score	Total NOS score	Risk of bias
Iwami et al. (2018) [3]	3	1	1	5 stars	Medium
Wang et al. (2018) [13]	3	1	3	7 stars	Low
Huang et al. (2019) [9]	3	1	2	6 stars	Medium
Peng et al. (2019) [5]	3	1	1	5 stars	Medium
Yildiz et al. (2019) [10]	3	2	2	7 stars	Low
Mathieu d’Argent E et al. (2020) [11]	4	1	2	7 stars	Low

### Quality of the evidence

The GRADE approach aims to evaluate the quality of the evidence for each major outcome. It also takes into consideration the results from the trial sequential analyses (see summary of findings for the main comparison, Table 3). For the primary outcomes of the clinical pregnancy rate, the quality of the RCT groups and subgroups was moderate, while the nonrandomized studies were low. For the live birth rate, the quality of the RCT groups and subgroups was high, while the nonrandomized studies were low. For OHSS, the quality was high. The quality of each secondary outcome is described in detail in Table 3.

**Table 3:**
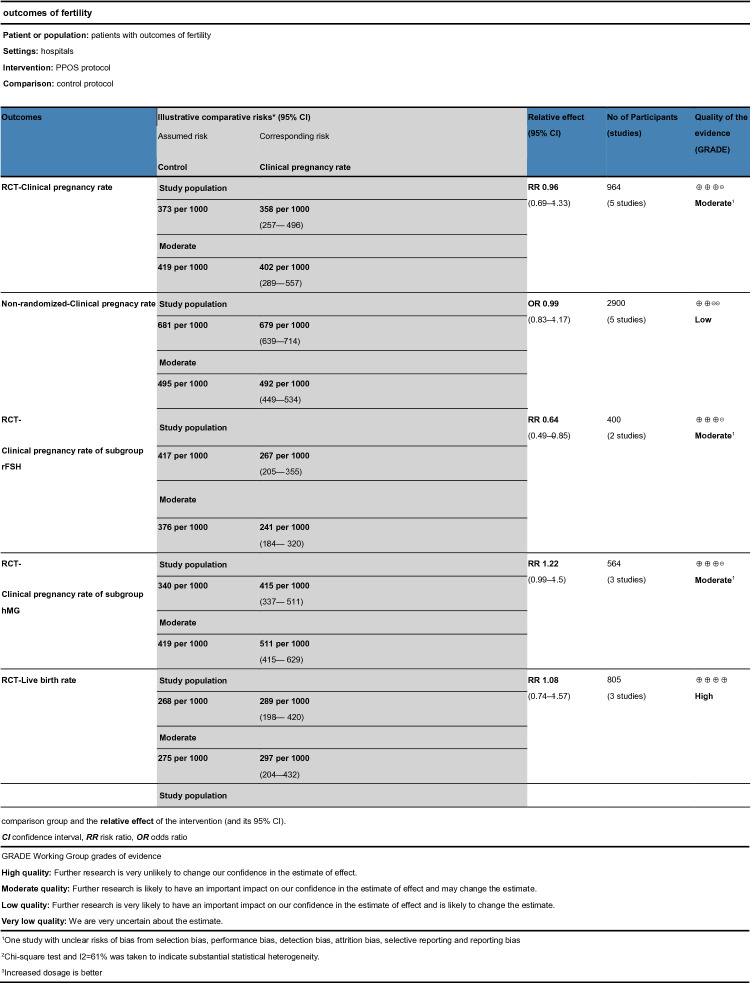
Summary of findings for the main comparison

### Primary outcomes

**Fig. 3:**
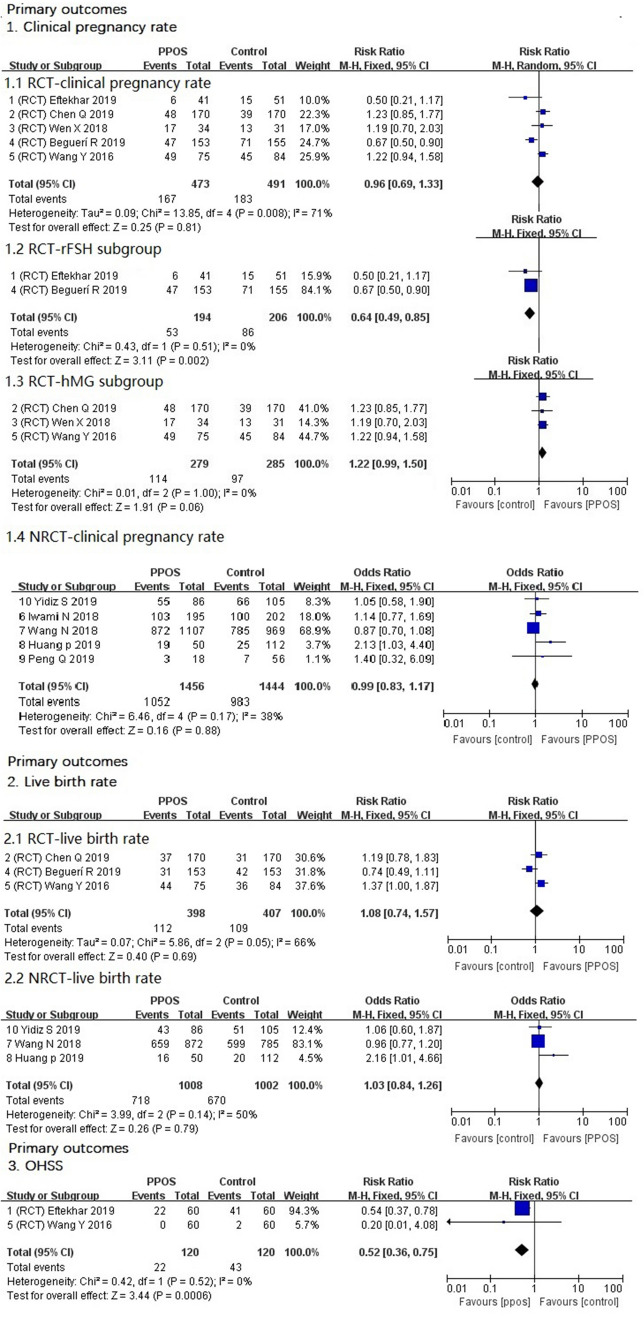
Forest plot of studies of primary outcomes

1. Clinical pregnancy rate
  Five RCTs showed that the clinical pregnancy rate with the PPOS protocol was not different from that with the control group [RR 0.96, 95% CI (0.69–1.33), *I*^2^ = 71%, *P* = 0.81].
  For *I*^2^ ≥ 70%, the heterogeneity was too large and not suitable for combined analysis. Analysis of the effectiveness of HMG versus recombinant FSH in women undergoing ovarian stimulation for IVF/ICSI demonstrated a significant difference in live birth rates [23, 24]. We performed subgroup analysis for the clinical pregnancy rate (primary outcome). Two RCTs in the rFSH subgroup showed that the PPOS protocol had a lower clinical pregnancy rate than the control group [RR 0.64, 95% CI (0.49–0.85), *I*^2^ = 0%], and the result was statistically significant (*P* = 0.002). Three RCTs showed that in the hMG subgroup, the PPOS protocol led to a higher clinical pregnancy rate than the control group [RR 1.22 95% CI (0.99–1.5), *I*^2^ = 0%, *P* = 0.06], and the difference was very close to being statistically significant.
  The results of five NRCTs did not show any significant difference in the clinical pregnancy rate between the two groups [RR 0.99, 95% CI (0.83–1.17), *I*^2^ = 38%, *P* = 0.88].
2. Live birth rate
  The live birth rates were not different between groups in three RCTs [RR 1.08, 95% CI (0.74, 1.57), *I*^2^ = 66%, *P* = 0.69]. Additionally, the results of one NRCT showed that there was no difference between the two groups [OR 1.03 95% CI 0.84–1.26), *I*^2^ = 50%, *P* = 0.79] (Fig. 3).
3. OHSS
  Only two RCTs described the incidence of OHSS, and the results showed that the PPOS protocol had a lower rate of OHSS [RR 0.52, 95% CI (0.36–0.75), *I*^2^ = 0%, *P* = 0.0006] (Fig. 3). The result was statistically significant.

### Secondary outcomes

**Fig. 4:**
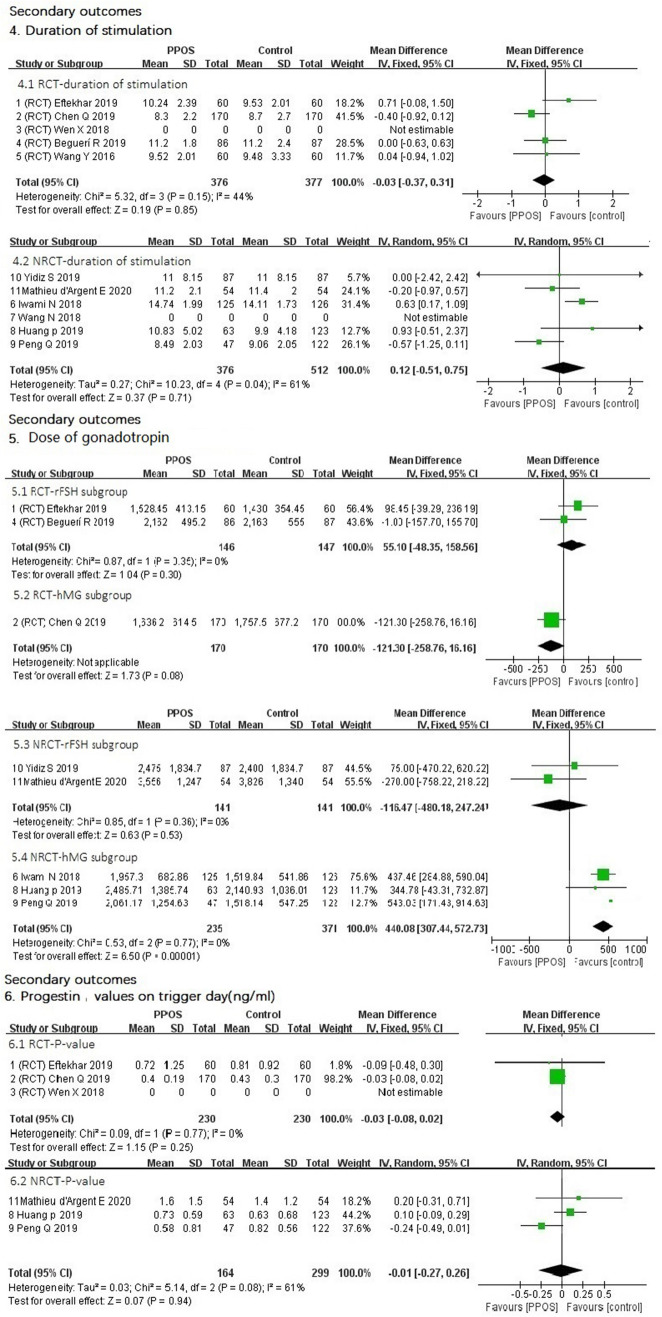
Forest plot of studies of secondary outcomes

**Fig. 5:**
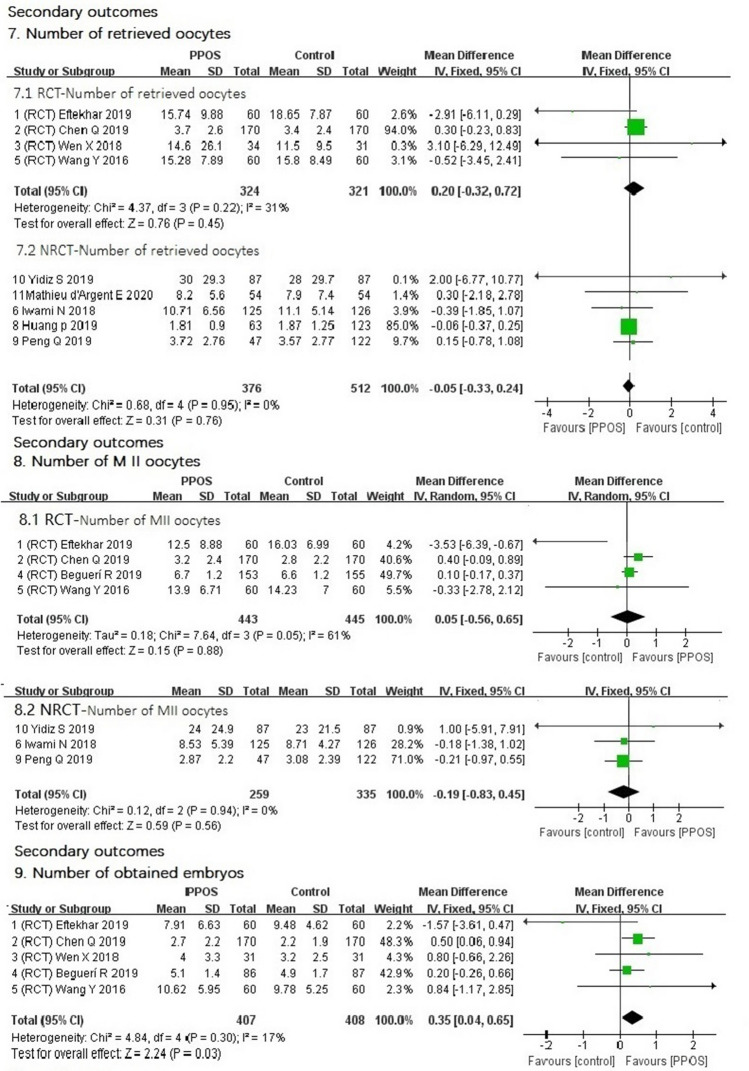
Forest plot of studies of secondary outcomes

**Fig. 6:**
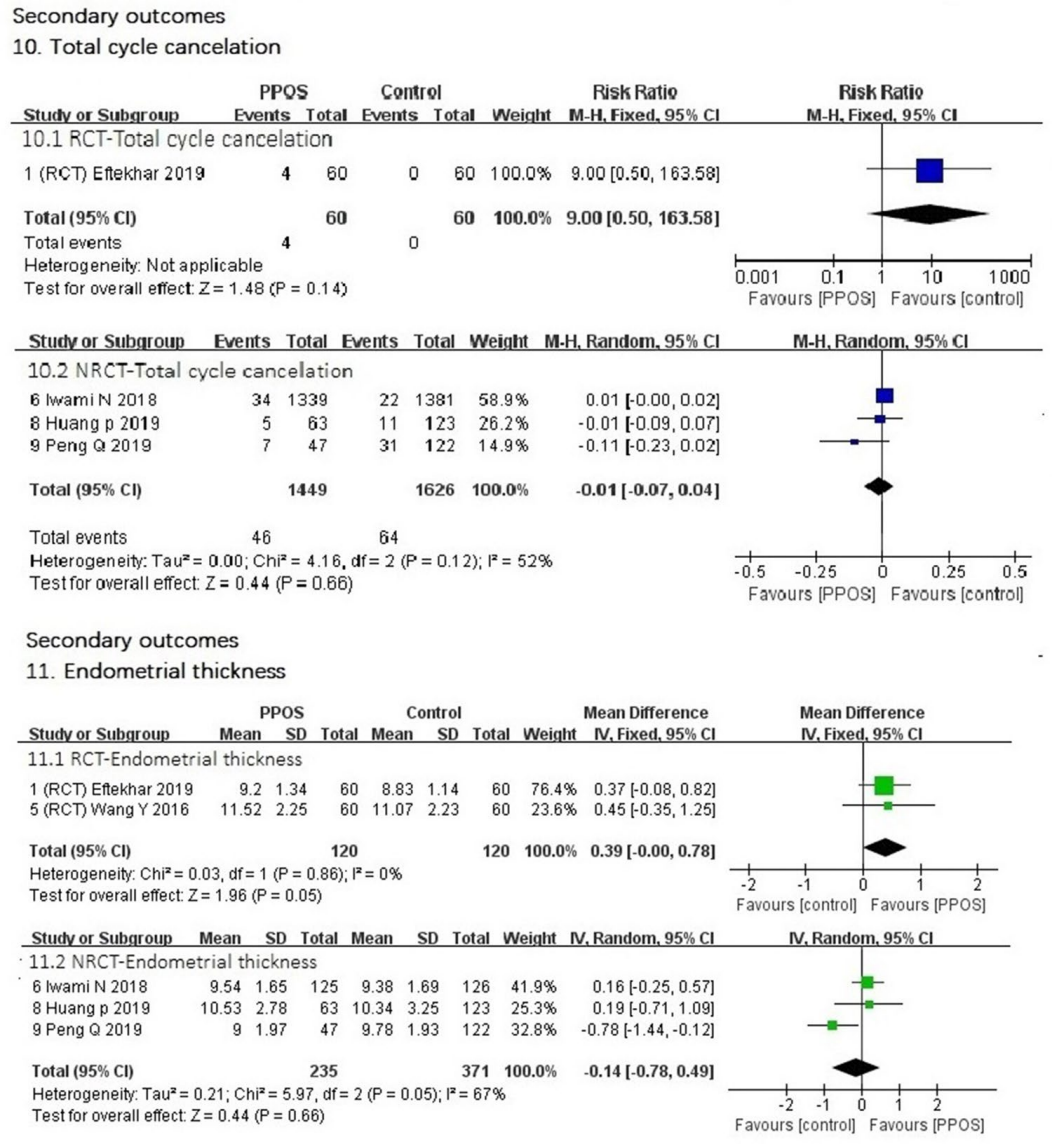
Forest plot of studies of secondary outcomes

4. Duration of stimulation (day)
  Data from both RCTs (MD 0.03 lower, 95% CI (− 0.37–0.31), *I*^2^ = 44%, *P* = 0.85) and nonrandomized trials (MD 0.12 higher, 95% CI (− 0.51–0.75), *I*^2^ = 61%, *P* = 0.71) showed that the duration of stimulation between the two groups was nearly the same. The slight difference was not statistically significant (Fig. 4).
5. Dose of gonadotrophin for injection (IU)
  We performed preplanned subgroup analysis of the dose of gonadotrophin for two different kinds of gonadotrophin. Two RCTs in the rFSH subgroup showed that the mean difference (MD) in dose for PPOS in the rFSH subgroup was 55.1 higher [95% CI (− 48.35–158.56), *I*^2^ = 0%, *P* = 0.30]. Only one RCT showed that the MD in dose of the PPOS protocol was 121.3 lower in the hMG subgroup [95% CI (− 258.76–16.16), *P* = 0.08]. These differences were not statistically significant. The results of NRCTs showed that the MD in the subgroup of rFSH was 116.47 lower [95% CI (− 480–247.24), *I*^2^ = 0%, *P* = 0.53]. NRCTs in the hMG subgroup showed that the MD for the PPOS protocol was 440.08 higher [95% CI (307.44, 572.73), *I*^2^ = 0%, *P* < 0.00001]. The difference was statistically significant (Fig. 4).
6. Progestin values on trigger day (ng/ml)
  Data from both RCTs [MD 0.03 lower, 95% CI (− 0.08–0.02), *I*^2^ = 0%, *P* = 0.25] and NRCTs [MD 0.01 lower 95% CI (− 0.27–0.26), *I*^2^ = 61%, *P* = 0.94] (Fig. 4) showed that the progestin values on the trigger day between the two groups were nearly the same. The slight difference was not statistically significant.
7. Number of retrieved oocytes
  Data from both RCTs [MD 0.2 higher, 95% CI (− 0.32–0.72), *I*^2^ = 31%, *P* = 0.45] and NRCTs [MD 0.05 lower 95% CI (− 0.33–0.24), *I*^2^ = 0%, *P* = 0.76] (Fig. 5) showed that the number of retrieved oocytes between the two groups was nearly the same.
8. Number of MII oocytes
  Data from either RCTs [MD 0.05 higher, 95% CI (− 0.56–0.65), *I*^2^ = 61%, *P* = 0.88] or NRCTs [MD 0.19 lower 95% CI (− 0.83–0.45), *I*^2^ = 0%, *P* = 0.56] (Fig. 5) showed that the number of MII oocytes between the two groups was nearly the same.
9. Number of obtained embryos
  Only the five RCTs (Fig. 5) had the date of the number of obtained embryos, and the result showed that the PPOS protocol had more obtained embryos [MD 0.35 higher 95% CI (0.04–0.65), *I*^2^ = 17%, *P* = 0.03]. The result was statistically significant.
10. Total cycle cancelation
  Data from both RCTs [95% CI (0.50–163.58), *P* = 0.14] and NRCTs [95% CI (− 0.07–0.04), *I*^2^ = 52%, *P* = 0.66] (Fig. 6) showed that there were no significant differences in the total cycle cancelation rates between the two groups.
11. Endometrial thickness (millimeter, mm)
  Data from RCTs showed that the endometrium was thicker with the PPOS protocol than with the control protocol [MD 0.39 mm, higher 95% CI (0.00–0.78), *I*^2^ = 0%, *P* = 0.05], and difference was statistically significant. Data from NRCTs (Fig. 6) showed that the endometrium was thinner with the PPOS protocol than with the control group [MD 0.14 mm lower 95% CI (− 0.78–0.49), *I*^2^ = 67%, *P* = 0.66], though the difference was not statistically significant.

## Discussion

The results of this meta-analysis showed that the PPOS protocol had more obtained embryos and a thicker endometrium than the control protocol, with a lower rate of OHSS. There were no significant differences in the live birth rate, duration of stimulation, progestin values on trigger day (ng/ml), number of retrieved oocytes, number of MII oocytes, or total cycle cancelation rates between the two groups.

In the rFSH subgroup, the clinical pregnancy rate was lower in the PPOS group than in the control group, and the result was statistically significant. Three RCTs showed that in the hMG subgroup, the clinical pregnancy rate of the PPOS protocol was higher than that of the control group, and the difference was near statistical significance (*P* = 0.06). The quality of the evidence (GRADE) was moderate. The results of the RCT of the rFSH/hMG subgroups showed that there was no significant difference in the dose of rFSH/hMG between the two groups, and the quality of the evidence (GRADE) was high. Only NRCTs in the hMG subgroup showed that the dose of hMG in the PPOS protocol was higher. Data from RCTs showed that the PPOS protocol had a thicker endometrium, and the quality of evidence was high with a significant difference. While NRCTs showed that the endometrium was thinner with the PPOS protocol, there was no significant difference, and the quality of evidence (GRADE) was low.

The prevalence of infertility is high around the world, and it is estimated that 1 out of 4 couples are infertile [25]. ART has developed quite rapidly over recent years, and there is still an unmet need for ovarian stimulation protocols with improved efficacy, safety, and convenience. New protocols, such as GnRH antagonist protocols and mild stimulation protocols, have been proposed over the last decade. Progestin-primed ovarian stimulation (PPOS) is also one of these new ovarian stimulation protocols. Some studies [26, 27] have suggested that compared with conventional ovarian stimulation methods, the PPOS protocol neither compromises neonatal outcomes of IVF newborns nor increases the prevalence of congenital malformations. This is the first meta-analysis to examine the effect of the PPOS protocol in ART. According to our review, the safety and effectiveness of PPOS are confirmed.

Poor ovarian response (POR) to ovarian hyperstimulation is one of the greatest challenges in assisted reproduction technology. According to the report from the Society for Assisted Reproductive Technology (SART) in 2018 in the USA, in women considered to be poor responders, there is fair evidence to support the recommendation that mild ovarian stimulation is cost-effective, although live birth rates are extremely low among both women undergoing the mild ovarian stimulation and those undergoing conventional IVF [28]. A retrospective study (Peng et al.) [5] showed no significant difference in the clinical pregnancy rates between the mild stimulation (12.5%) and PPOS groups (16.7%). The average numbers of oocytes and viable embryos and the live birth rates were comparable to those in the GnRH antagonist group. Although the PPOS protocol did not improve the clinical pregnancy rates of POR patients, it might be an option for personalized protocols.

In 2015, Dr. Kuang et al. [1] proposed the PPOS protocol such as medroxyprogesterone acetate (MPA) cotreatment with gonadotropin hMG during COS in IVF treatment. Several studies have suggested that progesterone in PPOS protocols may offer a variety of options such as medroxyprogesterone acetate (MPA), dydrogesterone [2–5, 28], or utrogestan [13, 29, 30]. In PPOS protocols, all of these options are sufficient to prevent an untimely LH rise. As DYG has been extensively used worldwide for the treatment of threatened miscarriage and recurrent miscarriage, DYG administration in PPOS protocols produces a comparable number of top-quality embryos and pregnancy outcomes compared with MPA [28]. However, further randomized controlled trials are needed to confirm this conclusion.

Recombinant follicle-stimulating hormone (rFSH) and human menopausal gonadotropin (uHMG) are widely used for controlled ovarian stimulation (COS). rFSH treatment results in a higher oocyte yield per cycle than human menopausal gonadotropin treatment [31, 32]. Different clinics choose different GN doses in PPOS protocols. From this meta-analysis, we conclude that there is no difference in the live birth rate. In the subgroup analysis, the hMG subgroup had a better clinical pregnancy rate, while the rFSH group had a lower clinical pregnancy rate than the control group. It may be suggested to choose hMG for COS in the PPOS protocol. A cost-effectiveness study [16] showed that PPOS protocols were cost-effective when freeze-only was planned for preimplantation genetic testing or fertility-preservation cycles, where a GnRH antagonist protocol would otherwise be used. In addition, this study cannot accurately specify drugs for PPOS protocols. More RCTs should be performed to evaluate the best drug candidates for individual infertile patients.

The strength of this meta-analysis lies in the strict methodology guided by PRISMA guidelines.

Additionally, the quality of the RCTs was evaluated using the Cochrane Handbook method as a way to enhance external validity. The quality of NRCTs was evaluated using the Newcastle–Ottawa Scale. Furthermore, we graded the certainty of the evidence for critical outcomes by GRADE-pro.

### Limitations of the review

Only five RCTs were included in our meta-analysis. The outcomes of NRCT by GRADE-pro were quite low. Furthermore, 6 of the 11 records included were from China. Progestin-primed ovarian stimulation (PPOS) was first proposed by the Yanping Kuang M.D. group in 2015. Over the last two years, many centers around the world have begun to choose PPOS.

## Conclusion

The PPOS protocol produces more obtained embryos and a thicker endometrium than the control group, with a lower rate of OHSS and equal clinical pregnancy rate, live birth rate, duration of stimulation, progestin value on trigger day (ng/ml), number of retrieved oocytes, number of MII oocytes, and total cycle cancelation rate. In the subgroup analysis, the hMG subgroup had a better clinical pregnancy rate, while the rFSH group had a lower clinical pregnancy rate than the control group. It may be suggested to choose hMG for COS in the PPOS protocol. More RCTs should be performed to evaluate the best ones for respective infertile patients.

## Data Availability

The data that support the findings of this study are available from the corresponding author upon reasonable request.
